# Medicare Beneficiary Receipt of Methadone by Drive Time to Opioid Treatment Programs

**DOI:** 10.1001/jamanetworkopen.2025.3099

**Published:** 2025-04-03

**Authors:** Jonathan Cantor, Helin G. Hernandez, Aaron Kofner, Julie Lai, Denis Agniel, Kosali I. Simon, Bradley D. Stein, Erin A. Taylor

**Affiliations:** 1 RAND, Santa Monica, California; 2 RAND, Arlington, Virginia; 3 O’Neill School of Public and Environmental Affairs, Indiana University, Bloomington; 4 RAND, Pittsburgh, Pennsylvania

## Abstract

**Importance:**

To combat the ongoing opioid crisis, policy makers and public health officials are developing novel policies to increase the availability of medications for opioid use disorder (OUD). An important question is to what extent geographic availability of opioid treatment programs (OTPs) is associated with treatment receipt. Understanding this association may help with developing additional policies to increase medication for OUD dispensing and improve population health outcomes.

**Objective:**

To quantify trends in dispensing methadone to Medicare beneficiaries based on proximity to an OTP.

**Design, Setting, and Participants:**

This cross-sectional study analyzed 2020 Medicare fee-for-service claims for methadone for beneficiaries with a recent diagnosis of OUD merged with drive times to OTP locations. Medicare beneficiaries enrolled in a Part D prescription plan and diagnosed with OUD in any of the 3 quarters before and during the 2020 quarter of interest were examined.

**Exposure:**

Drive time between the centroid of a beneficiary’s zip code and the closest OTP.

**Main Outcomes and Measures:**

Quarterly methadone receipt among Medicare beneficiaries with a recent OUD diagnosis was assessed using logistic regression models.

**Results:**

In 2020, there were 640 706 Medicare beneficiaries with a recent OUD diagnosis (mean [SD] age, 62.5 [13.5] years; 55.6% female; 65.5% residing in an urban locality at the time of diagnosis). Of these beneficiaries, 9.6% lacked an OTP within a 60-minute drive time. The probability of a beneficiary receiving methadone decreased as the drive time from an OTP increased. Specifically, in urban areas, the likelihood of methadone receipt decreased by a relative 54% from a mean of 5.29% (national interval, 4.27%-6.52%) for beneficiaries who lived within a 5-minute drive of an OTP to 2.39% (national interval, 1.92%-2.98%) for those who lived within a 15-minute drive from an OTP. For rural beneficiaries, the likelihood of methadone receipt decreased by a relative 27% from a mean of 3.42% (national interval, 2.73%-4.28%) for a 5-minute drive time to 2.39% (national interval, 1.92%-2.98%) for a 15-minute drive time. Evidence of a threshold effect at a drive time of 20 minutes was observed for methadone receipt, after which the rate slowed and was similar between urban and rural beneficiaries.

**Conclusions and Relevance:**

These findings suggest that the likelihood of methadone receipt may vary based on proximity to OTP facilities. Where OTPs are located may be a contributor to whether an individual receives methadone treatment.

## Key Points

**Question:**

What is the association of opioid treatment program availability with dispensation of methadone, a medication for opioid use disorder, for Medicare beneficiaries with a recent diagnosis of opioid use disorder?

**Findings:**

In this cross-sectional study of 640 706 Medicare beneficiaries, drive time increases from 5 to 15 minutes were associated with a 54% relative reduction in the likelihood of receiving methadone, with the respective likelihoods dropping from 5.29% to 2.39%, on average. For rural residents, drive time increases from 5 to 15 minutes were associated with a 27% relative reduction in the likelihood of receiving methadone, with respective likelihoods dropping from 3.42% to 2.49%, on average.

**Meaning:**

These findings suggest that future policy interventions aimed at improving treatment accessibility and methadone uptake might consider strategically increasing the availability of opioid treatment programs in underserved areas.

## Teaser

This cross-sectional study examines the association between drive time to an opioid treatment program and receipt of methadone by Medicare beneficiaries with opioid use disorder.

## Introduction

The opioid crisis is an ongoing and urgent national public health problem,^1,2^ with growing concerns about the number of older adults (aged ≥65 years) diagnosed with opioid use disorder (OUD).^3^ Medicare, which provides health care coverage to individuals aged 65 years or older and individuals with disabilities aged 64 years or younger, covered more than 1 million beneficiaries diagnosed with OUD in 2021.^4^ Older adults may be more vulnerable to opioid overdose due to the physiologic changes of aging and increased chronic medical disease burden,^5^ and fatal overdoses among people aged 65 years or older quadrupled between 2002 and 2021.^6^

Three forms of medications for OUD (MOUDs) exist, with differing dispensing regulations. Methadone traditionally is provided during in-person visits at an opioid treatment program (OTP). In contrast, buprenorphine and naltrexone do not have the same strict regulatory constraints and can be prescribed by any licensed practitioner. According to the Substance Abuse and Mental Health Services Administration, there were 1952 certified OTPs as of November 2023.^7^ These programs were more commonly located in urban than rural areas,^8,9,10^ which may be associated with barriers to the creation and location selection of OTPs.^11,12^ A combination of local zoning rules and politician attitudes limit the opening of OTPs, as well as how many patients they can treat.^13^ Rural OTPs also face substantial financial challenges in their operations.^14^ In addition, buprenorphine prescribers tend to locate near OTPs.^15^ The geographic maldistribution of OTPs raises serious concerns that many individuals may be unable to access MOUD services.^16^

The lack of geographic access to OTPs may be exacerbated by other payment-based barriers to receiving OUD treatment. Before 2020, Medicare did not cover methadone for the treatment of OUD. The Substance Use Disorder Prevention That Promotes Opioid Recovery and Treatment for Patients and Communities Act of 2018 created a new Medicare Part B benefit that pays for OUD treatment,^17^ including methadone, provided by OTPs. The policy took effect in January 2020 and was expected to drastically transform the treatment landscape for Medicare beneficiaries with OUD.^18^ Early trends in 2020 showed a large increase in OTP acceptance of Medicare as a form of payment,^19,20^ methadone use among Medicare beneficiaries,^21,22^ and changes in county-level accessibility of MOUD services.^23^

Previous research has used distance-based measures to calculate the geographic accessibility of MOUDs at OTPs and other health care practitioners and facilities.^9,10,24,25,26,27,28^ It is well established that residents of rural counties have longer drive times to the nearest OTP compared with residents of urban counties.^9,10,29,30^ However, no national studies to date have assessed how geographic access to an OTP may be associated with receipt of methadone by patients with OUD and whether this association varies by urbanicity.^31^

Given that methadone generally requires in-person visits at an OTP, an investigation into the association between drive time to an OTP and methadone receipt may provide important insights into the extent to which distance influences access to MOUDs. We investigated 2 primary research questions related to the possible association between geographic access to an OTP and methadone receipt among Medicare beneficiaries diagnosed with OUD.

First, we asked whether a beneficiary’s drive time to an OTP is associated with the likelihood of methadone receipt. On the basis of existing literature,^32,33^ we hypothesized that longer drive time to the closest OTP would be associated with lower probability of methadone receipt. Second, we asked whether there is a drive time threshold effect on methadone receipt (ie, a nonlinear association between proximity to an OTP and methadone receipt). On the basis of previous studies,^34^ we hypothesized that there would be a nonlinear association between drive time and methadone receipt; however, we did not have evidence to support a hypothesis for the specific drive time distance at which the threshold effect would occur. Within the second research question, we were particularly interested about whether the drive time was different by urbanicity. The travel time to health care for rural and urban individuals has changed over time.^35^ Drive time distance to a practitioner or facility is a measure of accessibility to health care services. In contrast, the urbanicity of a community encompasses sociodemographic and geographic characteristics that influence health care receipt. Holistically, the answers to these questions may enhance our understanding of how Medicare beneficiaries receive methadone and potentially inform policy changes to improve access.

## Methods

This cross-sectional study was determined to be exempt from local ethics review and informed consent by the RAND’s Institutional Review Board as it did not involve human participants. This study followed the Strengthening the Reporting of Observational Studies in Epidemiology (STROBE) reporting guideline for cross-sectional studies.

### OTP and Medicare Claims Data

On May 3, 2023, we downloaded a database containing all OTPs in the US, maintained by the Centers for Medicare & Medicaid Services, and derived from information in the Provider Enrollment, Chain, and Ownership System.^36^ We geocoded all OTPs at the address level using ArcGIS Pro 3.1 (Esri).

We used Medicare Part B carrier files for 2019 and 2020 to identify beneficiaries with a recent OUD diagnosis, which we defined as any diagnosis of OUD in the 3 quarters before and including the quarter of interest. To identify a diagnosis of OUD, we used *International Classification of Diseases, Ninth Revision* and *International Statistical Classification of Diseases, Tenth Revision* codes. The full list of codes is provided in eAppendix 1 in Supplement 1. We included beneficiaries in our sample if they had a recent OUD diagnosis and were enrolled in a Medicare Part D prescription drug plan in the quarter of interest.

Although data on race and ethnicity were available in the Master Beneficiary Summary File, we decided not to use these data in our analysis for 2 reasons. First, the Master Beneficiary Summary File race and ethnicity information is less reliable compared with alternatives such as geocoded race and ethnicity categories derived from Medicare Bayesian Improved Surname Geocoding algorithm, which we did not have access to. Second, our study was strictly focused on the geographic accessibility of treatment resources. Therefore, while we had the race and ethnicity data, our decision not to use it was based on considerations of data reliability and the specific aims of our research.

We identified methadone dispensing by OTPs in the Part B carrier files using Health Care Common Procedural Coding System codes and buprenorphine and naltrexone claims in both the carrier and Part D Event data using Health Care Common Procedural Coding System and National Drug Code codes. We measured 4 MOUD receipt outcomes in each quarter of 2020: any buprenorphine, any methadone, any naltrexone, and any (of those 3) MOUDs. Methadone receipt measures are reported herein. Buprenorphine, naltrexone, and any MOUD receipt results are reported in Supplement 1 and are intended as hypothesis generating rather than as inference. It is important to note that our study focused solely on rates of receipt and did not quantify measures of initiation or retention.

### Drive Time Measures

We used ArcGIS Pro 3.1 and ArcGIS StreetMap Premium (2022) to geocode all OTPs listed by the Centers for Medicare & Medicaid Services based on their street address information. We then used the Make OD Cost Matrix Analysis Layer toolset within the Network Analyst toolbox to create a drive time measure between all origins (US zip code centroids) and all destinations (geocoded OTP facilities). This tool uses a street network file to calculate the fastest drive time path between 2 points. We collapsed the data to the zip code level and created a drive time variable, which represents the shortest drive time between the centroid and nearest OTP facility for each US zip code.

### Statistical Analysis

To evaluate both research questions, we fit generalized additive logistic regression models^37,38^ (1 for each receipt outcome and quarter) (detailed specification provided in eAppendix 2 in Supplement 1). Quarter was used as the temporal unit of analysis to show changes over time, since 2020 was the first year methadone was reimbursed by Medicare and we wanted to be consistent with previous work on methadone receipt during the COVID-19 pandemic.^21,39^ For computational reasons, we estimated the regressions separately for each quarter. These models incorporated the drive time measure previously described based on the beneficiary’s zip code of residence and whether their Federal Information Processing Standards code was classified as rural or urban using National Center of Health Statistics definitions. Metropolitan counties include large metropolitan statistical areas with populations of 1 million people or more, medium metropolitan statistical areas with populations of 250 000 to 999 999 people, and small metropolitan statistical areas with populations of less than 250 000 people. In contrast, nonmetropolitan areas include micropolitan urban clusters with populations between 10 000 and 49 999 people, as well as noncore counties not part of micropolitan statistical areas.^40^ Counties classified as metropolitan were considered urban, and counties classified as nonmetropolitan were considered rural.

To answer the second research question of whether there is a drive time threshold effect on methadone receipt, we captured varying trends by drive time across levels of urbanization by incorporating a smoothed 2-way interaction term between the drive time measure and location (urban vs rural). Although rurality and drive time to an OTP may be related, we believed that these are 2 distinct constructs that may influence receipt of MOUD differently. While drive time distance specifically measures accessibility to health care services, rurality captures broader sociodemographic and geographic characteristics that may influence health care receipt. For example, in urban areas, a 30-minute drive might be perceived as a considerable deterrent despite having access. This perception may be due to competing demands on time (eg, work, traffic, childcare). However, in rural settings, driving long distances may be more common. Here, other factors such as infrastructure limitations, lack of public transportation, and cultural attitudes toward health care may play more substantial roles in MOUD receipt. Adding the interaction term between drive time and rurality to the model increases flexibility to capture varying associations between drive time and MOUD receipt that differ by rurality. To further support the inclusion of the interaction term, we fit models with and without the interaction term. Akaike information criterion and bayesian information criterion calculations indicated that the interaction term improved model fit.

A beneficiary’s state of residence was also taken into account via fixed effects, as OTPs may respond differently to the federal change in Medicare policy due to different state-level policies.^12^ To improve model stability, we standardized the drive time measure to have a mean of 0 and SD of 1, using the mean (SD) of 23.15 (25.57) minutes from the full dataset.

While our regression models estimated the state-specific probability of methadone receipt, we aimed to highlight national averages. To estimate national probabilities, we first obtained the state-specific point estimates and the respective 95% CIs for these probabilities. We then calculated the national point estimate by averaging the state-specific probability point estimates. Similarly, we derived the national interval of plausible values for methadone receipt probabilities by averaging the lower and upper bounds of the state-specific confidence intervals. These aggregated estimates are presented herein, while those for buprenorphine, naltrexone, and any MOUD receipt are presented in eTable 2 and eFigure 3 in Supplement 1.

All analyses were completed using SAS, version 9.4 (SAS Institute Inc), and R, version 4.3.2 (R Foundation). Significant differences were when the national interval bounds did not overlap.

## Results

### Descriptive Analysis

We identified a total of 640 706 Medicare beneficiaries with a recent OUD diagnosis in 2020 (mean [SD] age, 62.5 [13.5] years; 55.6% female and 44.4% male; 65.5% resided in an urban locality). The number of beneficiaries with a recent OUD diagnosis decreased slightly throughout 2020 (Table 1; eTable 1 in Supplement 1). In 2020, 4.1% of these beneficiaries received methadone. The rates of methadone receipt was highest the closer a beneficiary lived to an OTP and declined with longer drive times. Notably, while the number of beneficiaries with OUD decreased throughout 2020, the receipt of methadone increased across 2020 conditional on drive time (from 3.0% in quarter 1 to 4.3% in quarter 4 for <15 minute drive time) (Table 1).

**Table 1.** Empirical National Methadone Receipt Likelihood by Quarter and Drive Time From Nearest OTP Based on a Medicare Beneficiary’s Zip Code Centroid

Drive time, min	Beneficiaries with methadone receipt, No. (%)
Q1 (n = 478 820)	
<15 min	14 516 (3.0)
15-30 min	2881 (0.6)
30-60 min	1386 (0.3)
>60 min	611 (0.1)
Q2 (n = 459 909)	
<15 min	15 171 (3.3)
15-30 min	3195 (0.7)
30-60 min	1532 (0.3)
>60 min	684 (0.1)
Q3 (n = 447 204)	
<15 min	18 159 (4.1)
15-30 min	3774 (0.8)
30-60 min	1737 (0.4)
>60 min	798 (0.2)
Q4 (n = 428 984)	
<15 min	18 405 (4.3)
15-30 min	3878 (0.9)
30-60 min	1771 (0.4)
>60 min	835 (0.2)

Abbreviations: OTP, opioid treatment program; Q, quarter.

**Figure 1.**
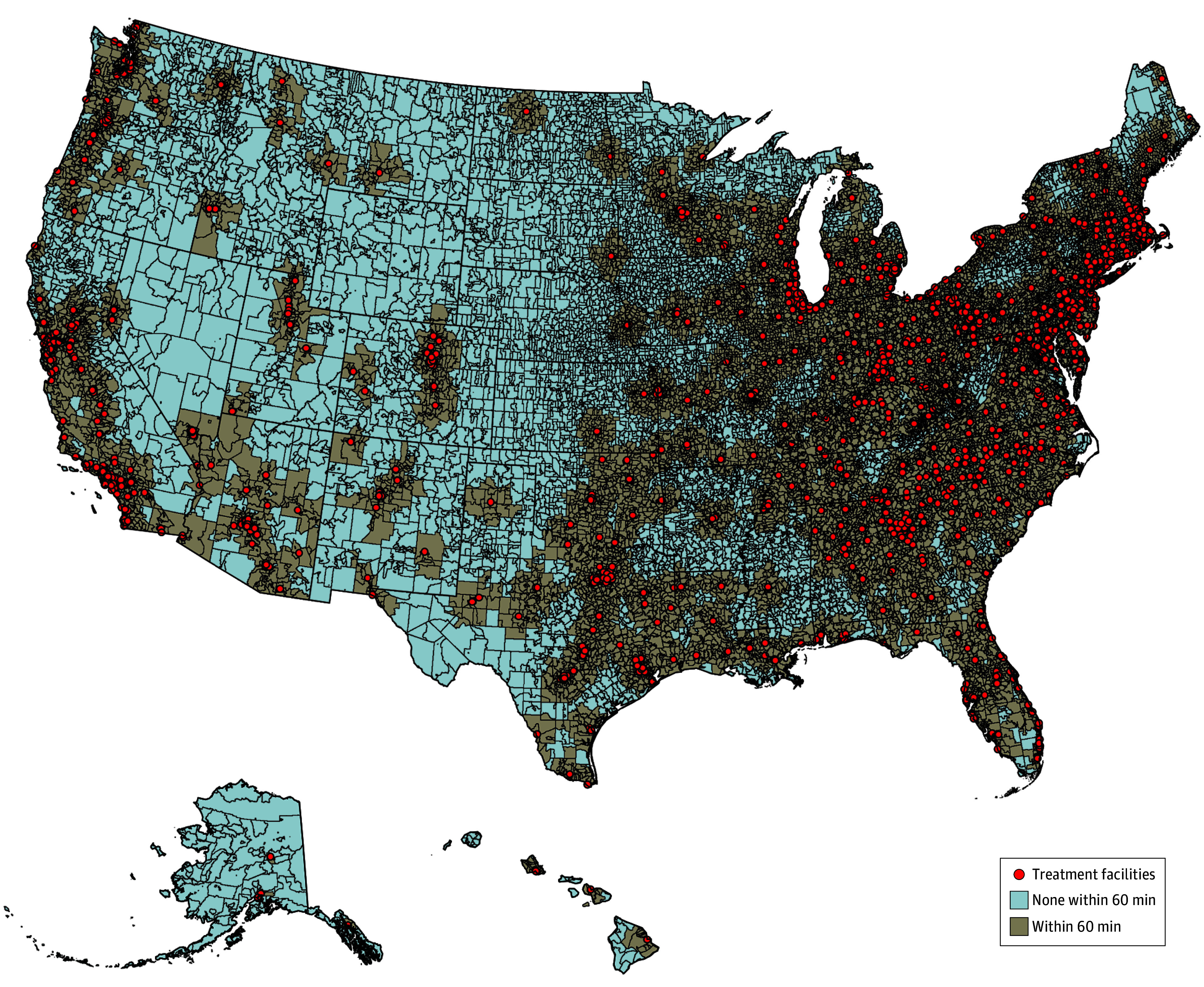
Geographic Variation in Availability of an Opioid Treatment Program Within a 60-Minute Drive Time Dots represent opioid treatment programs that are listed with the Centers for Medicare & Medicaid Services.

Figure 1 shows the variation in availability of an OTP being within a 60-minute drive from the centroid of a beneficiary’s zip code. eFigures 1 and 2 in Supplement 1 show versions of the map with a 15-minute and 30-minute drive time, respectively. Of a total of 31 985 zip codes, 76.2% (49.6% of Medicare beneficiaries diagnosed with OUD) lacked an OTP within a 15-minute network drive time, 52.6% (25.2% of Medicare beneficiaries diagnosed with OUD) lacked an OTP within a 30-minute drive time, and 25.0% (9.6% of Medicare beneficiaries diagnosed with OUD) lacked an OTP within a 60-minute drive time.

### Regression Analysis

**Figure 2.**
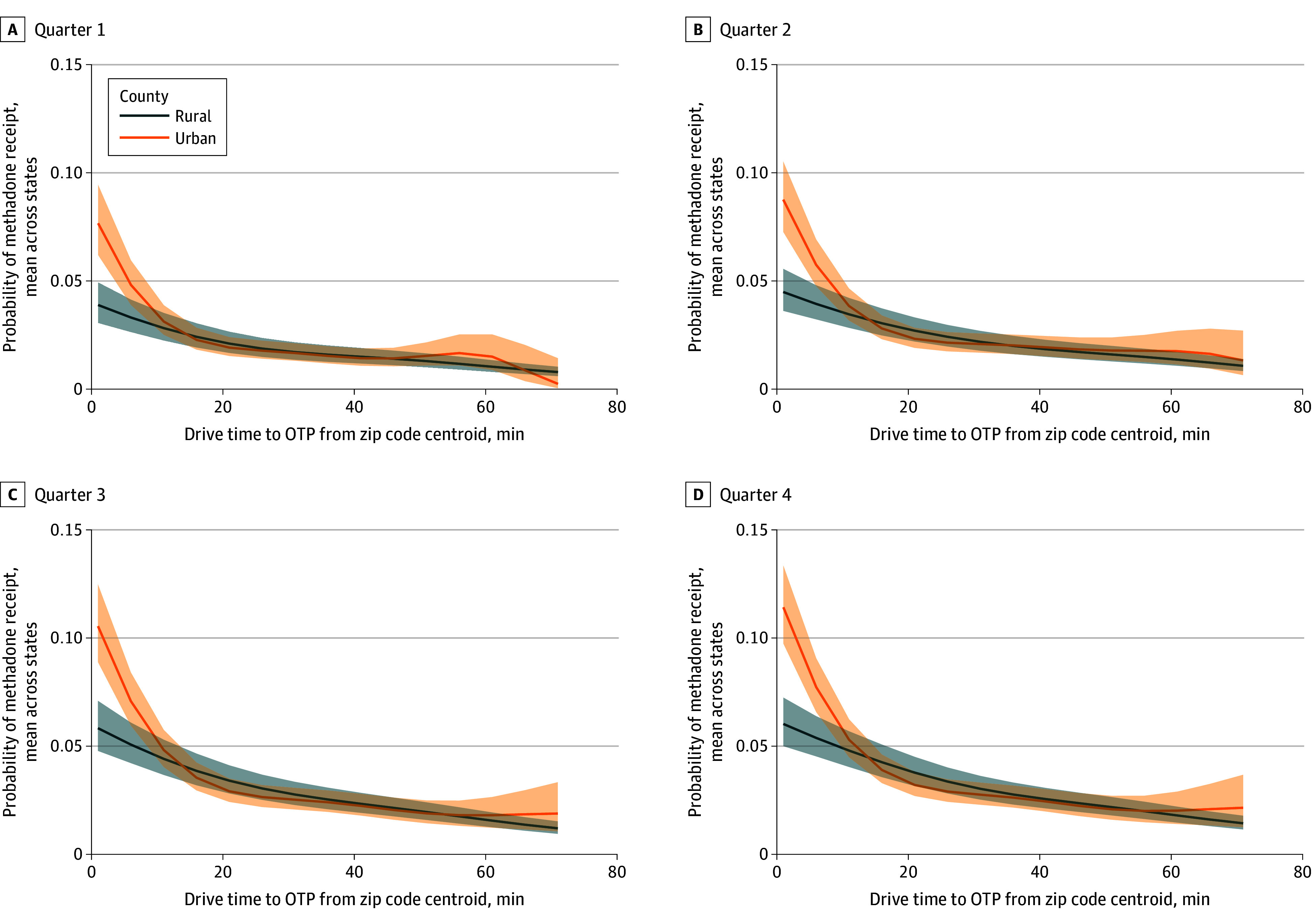
Estimated Probabilities of Methadone Receipt Based on Drive Time in Each Quarter of 2020 Shaded areas indicate the national interval.

Figure 2 compares drive time to an OTP with methadone receipt. As drive time increased, the likelihood of methadone receipt decreased, particularly among urban beneficiaries. Specifically, the likelihood of methadone receipt for urban residents relatively decreased by 54% from a mean of 5.29% (national interval, 4.27%-6.52%) for a 5-minute drive time to 2.39% (national interval, 1.92%-2.98%) for a 15-minute drive time (Table 2). In contrast, the likelihood of methadone receipt for rural residents relatively decreased by 27% from a mean of 3.42% (national interval, 2.73%-4.28%) for a 5-minute drive time to 2.49% (national interval, 1.98%-3.12%) for a 15-minute drive time. Methadone receipt was similar in urban and rural beneficiaries for all drive times to an OTP longer than 15 minutes. Figure 2 also shows a threshold effect in the receipt of methadone. Specifically, at drive times less than 20 minutes to an OTP, the likelihood of methadone receipt decreased, after which the rate of decrease became less pronounced. This methadone threshold effect was consistent across both urban and rural counties.

**Table 2.** National Likelihood of Methadone Receipt by Quarter and Drive Time From the Nearest OTP Based on a Medicare Beneficiary’s Zip Code Centroid

Drive time	Estimated likelihood across states, mean (national interval)	
	Rural beneficiaries with methadone receipt	Urban beneficiaries with methadone receipt
5 Min		
Q1	3.42 (2.73-4.28)	5.29 (4.27-6.52)
Q2	4.05 (3.31-4.94)	6.26 (5.19-7.53)
Q3	5.21 (4.33-6.27)	7.67 (6.45-9.10)
Q4	5.5 (4.62-6.54)	8.36 (7.12-9.79)
15 Min		
Q1	2.49 (1.98-3.12)	2.39 (1.92-2.98)
Q2	3.12 (2.55-3.82)	2.96 (2.44-3.59)
Q3	3.96 (3.28-4.77)	3.73 (3.11-4.47)
Q4	4.36 (3.65-5.19)	4.11 (3.47-4.86)
30 Min		
Q1	1.74 (1.38-2.19)	1.69 (1.33-2.14)
Q2	2.23 (1.81-2.74)	2.09 (1.70-2.58)
Q3	2.81 (2.32-3.41)	2.54 (2.09-3.09)
Q4	3.09 (2.58-3.69)	2.78 (2.31-3.34)
60-Min		
Q1	1.06 (0.82-1.37)	1.57 (0.96-2.58)
Q2	1.39 (1.10-1.75)	1.76 (1.16-2.66)
Q3	1.59 (1.28-1.98)	1.8 (1.24-2.60)
Q4	1.83 (1.49-2.25)	2.01 (1.41-2.84)

Abbreviations: OTP, opioid treatment program; Q, quarter.

Table 3 and eTable 3 in Supplement 1 show the empirical state-specific methadone and MOUD receipt rates, respectively, for Medicare beneficiaries in 2020. Both tables show notable geographic disparities. Methadone receipt showed wide variation, with a handful of states having a rate greater than 10%, including Connecticut (18.4%), Maine (11.7%), Maryland (17.4%), Massachusetts (15.5%), and Rhode Island (17.0%), and other states having rates of less than 1%, including Idaho (0.5%), Nebraska (0.9%), and Mississippi (0.9%).

**Table 3.** Empirical State-Specific Methadone Receipt Among Medicare Beneficiaries, 2020^a^

State	Beneficiaries with methadone receipt, No. (%)
Alabama	515 (4.5)
Alaska	30 (2.1)
Arizona	432 (2.6)
Arkansas	72 (1.2)
California	5051 (8.0)
Colorado	279 (3.0)
Connecticut	1406 (18.4)
Delaware	207 (3.8)
District of Columbia	106 (6.1)
Florida	595 (1.1)
Georgia	476 (2.8)
Hawaii	91 (6.7)
Idaho	22 (0.5)
Illinois	601 (3.9)
Indiana	444 (3.1)
Iowa	103 (3.1)
Kansas	49 (1.0)
Kentucky	571 (3.1)
Louisiana	193 (1.6)
Maine	551 (11.7)
Maryland	2912 (17.4)
Massachusetts	3344 (15.5)
Michigan	1520 (5.9)
Minnesota	403 (4.3)
Mississippi	108 (0.9)
Missouri	212 (2.1)
Montana	77 (3.9)
Nebraska	16 (0.9)
Nevada	186 (2.5)
New Hampshire	316 (7.9)
New Jersey	801 (4.4)
New Mexico	233 (4.2)
New York	2051 (7.4)
North Carolina	786 (3.4)
North Dakota	NA^b^
Ohio	517 (2.7)
Oklahoma	194 (1.1)
Oregon	424 (4.2)
Pennsylvania	1152 (3.9)
Rhode Island	336 (17.0)
South Carolina	395 (5.2)
South Dakota	NA^c^
Tennessee	505 (3.1)
Texas	465 (1.4)
Utah	106 (1.9)
Virginia	663 (6.0)
Washington	1022 (6.3)
West Virginia	272 (4.9)
Wisconsin	490 (7.0)
Wyoming	NA^b^

Abbreviation: NA, not available.

^a^ Receipt at either an opioid treatment program or covered by Medicare Part D at pharmacies for beneficiaries with a Part D plan.

^b^ Counts less than 11 were suppressed per Centers for Medicare & Medicaid Services privacy protocol.

^c^ No beneficiary with opioid use disorder was treated.

## Discussion

This cross-sectional study provides additional evidence that the opioid crisis remains a pressing national public health issue, with older adults increasingly affected. The introduction of Medicare payment for OTP care was hailed as a transformative event in the treatment of OUD for older adults,^18^ and OTPs quickly began to accept Medicare payments.^21,22^ This policy change also opened an exciting new opportunity to understand the association between travel distance and MOUD use using location-based data. Focusing on Medicare beneficiaries is particularly crucial, as 21% who did not receive treatment for past-year substance use disorder cited logistical barriers, such as lack of transportation, as a reason.^41^

We found that the drive time needed to reach an OTP may serve as a major barrier to receiving methadone, which must be dispensed in-person frequently.^42,43^ To our knowledge, this study is the first to link national health care claims data of Medicare beneficiaries with OUD with drive time measures to OTPs. We found that 49.6% of Medicare beneficiaries with OUD lacked access to an OTP within a 15-minute drive time and that 9.6% lacked access to an OTP within a 60-minute drive time. Previous research has found that even after a 10-mile Euclidean distance, the likelihood of methadone treatment retention decreases.^44^ Given our results, the maldistribution of OTPs relative to Medicare beneficiaries with an OUD diagnosis may contribute to reduced rates of methadone receipt among Medicare beneficiaries. Our findings may also contribute to policies designed to prioritize certain geographic areas for the expansion of methadone access to reduce travel times to a health care practitioner or facility.^31^

Our findings highlight the differential effects that the proximity and urbanicity of a community may have on receipt of methadone. First, the likelihood of methadone receipt decreased as drive time to an OTP increased and was more pronounced for urban residents, dropping a relative 54% for those with a 5-minute vs 15-minute drive time. On the other hand, we found that rural residents only experienced a relative 27% decrease over the same drive time. Beyond a 15-minute drive time, methadone receipt rates were similar for both urban and rural residents. Evidence of a threshold effect was observed for methadone receipt at a 20-minute drive time, after which the rate of decrease became less pronounced.

These findings have several important policy implications. The significant decrease in methadone receipt with increased drive time reiterates the need for more strategically located OTPs, especially in underserved areas. Policies aimed at reducing zoning and regulatory barriers to opening new OTPs may enhance access. In addition, future research should explore the role that telehealth services might serve in ensuring that health care practitioners and facilities in rural counties are equipped to offer MOUDs. The use of telehealth may help bridge the treatment gap in regions where OTPs are scarce.

We also found drastic differences in methadone receipt at the state level. Our results pinpointed states with high methadone receipt rates to observe successful strategies and identify states with low methadone receipt rates to implement policies that might boost receipt. While strategically located OTPs may improve methadone receipt, improving receipt to other MOUDs is equally important. In certain states, such as West Virginia before 2018,^45^ the lack of Medicaid coverage for OTPs may contribute to the reliance on alternative medications such as buprenorphine. These findings suggest that alternative approaches beyond strategically placing OTPs may improve receipt of other MOUDs.

### Limitations

This study is subject to some constraints that limit our inferences. First, the study used the centroid of a beneficiary’s zip code as their location to calculate their drive time to an OTP. A more precise measure of travel time would use the beneficiary’s home location. Second, OUD may have been underdiagnosed in health care claims data for Medicare beneficiaries. Therefore, our analyses may have underestimated the need for MOUDs among Medicare beneficiaries, as well as the travel barriers to MOUD access. Third, we had no clinical information regarding patient status, use of MOUDs, clinical improvement in response to MOUDs, or quality of care. Fourth, we used county-level measures for urbanicity, which may have missed important variation at a more granular level of geography, such as census tract^46^ or census block groups.^47^ Future work should conduct similar research if data at a more granular level of geography are available. Fifth, this study strictly focused on Medicare fee-for-service beneficiaries; thus, the results may not be generalizable to other patient populations or insurers. Sixth, the focus of this cross-sectional study was to explore associations between drive time to an OTP and MOUD receipt; therefore, the results should be interpreted as associations and not causal relationships. Seventh, there were other confounding factors that we were unable to adjust for, including the impact of the COVID-19 pandemic on medication receipt and race and ethnicity of the patient. It has been well established that the COVID-19 pandemic reduced MOUD initiation^48^ and that disparities exist in MOUD receipt based on patient race and ethnicity.^49^ Eighth, our study only focused on methadone receipt for which there were few recipients in some states, which may have influenced the robustness of our findings. Finally, the study did not seek to understand changes in methadone initiation and retention. Future work should explore whether travel time to an OTP is associated with the likelihood of methadone initiation and retention.

## Conclusions

In this cross-sectional study of Medicare fee-for-service beneficiaries with a recent OUD diagnosis, we quantified the association between geographic access to OTPs and methadone receipt. Reducing existing urban-rural disparities in geographic methadone access may substantially alleviate the burden of OUD for older adults and improve public health outcomes. As the opioid crisis continues to evolve, policy makers must create and enact targeted policies and interventions to ensure that all individuals, regardless of location, have access to life-saving OUD treatment.

## Notes

Supplement 1. **eFigure 1.** Geographic Variation in Availability of an OTP Within a 15-Minute Drive **eFigure 2.** Geographic Variation in Availability of an OTP Within a 30-Minute Drive **eFigure 3.** Estimated Probabilities of MOUD Use Based on Drive Time in Each Quarter of 2020 **eTable 1.** Empirical National MOUD Receipt Likelihood by Quarter and Drive Time (Minutes) From Nearest OTP Based on a Beneficiary’s Zip Code Centroid **eTable 2.** National Likelihood of Any MOUD Receipt by Quarter and Drive Time (Minutes) From the Nearest OTP Based on a Beneficiary’s Zip Code Centroid **eTable 3.** Empirical State-Specific MOUD in 2020
eAppendix 1.

eAppendix 2.

Supplement 2.
Data Sharing Statement
