# Supplementary material for: Medicare Beneficiary Receipt of Methadone by Drive Time to Opioid Treatment Programs
Source: JAMA Netw Open. 2025 Apr 3;8(4):e253099. doi: 10.1001/jamanetworkopen.2025.3099 (PMC11969284; doi:10.1001/jamanetworkopen.2025.3099)
Supplement: Supplement 1. — eFigure 1. Geographic Variation in Availability of an OTP Within a 15-Minute Drive eFigure 2. Geographic Variation in Availability of an OTP Within a 30-Minute Drive eFigure 3. Estimated Probabilities of MOUD Use Based on Drive Time in Each Quarter of 2020 eTable 1. Empirical National MOUD Receipt Likelihood by Quarter and Drive Time (Minutes) From Nearest OTP Based on a Beneficiary’s Zip Code Centroid eTable 2. National Likelihood of Any MOUD Receipt by Quarter and Drive Time (Minutes) From the Nearest OTP Based on a Beneficiary’s Zip Code Centroid eTable 3. Empirical State-Specific MOUD in 2020 eAppendix 1. eAppendix 2. [file jamanetwopen-e253099-s001.pdf]

Supplemental Online Content

Cantor J, Hernandez HG, Kofner A, et al. Medicare beneficiary receipt of methadone by drive time to opioid treatment programs. *JAMA Netw Open*. 2025;8(4):e253099. doi:10.1001/jamanetworkopen.2025.3099

eFigure 1. Geographic Variation in Availability of an OTP Within a 15-Minute Drive

eFigure 2. Geographic Variation in Availability of an OTP Within a 30-Minute Drive

eFigure 3. Estimated Probabilities of MOUD Use Based on Drive Time in Each Quarter of 2020

eTable 1. Empirical National MOUD Receipt Likelihood by Quarter and Drive Time (Minutes) From Nearest OTP Based on a Beneficiary's Zip Code Centroid

eTable 2. National Likelihood of Any MOUD Receipt by Quarter and Drive Time (Minutes) From the Nearest OTP Based on a Beneficiary's Zip Code Centroid

eTable 3. Empirical State-Specific MOUD in 2020

eAppendix 1

eAppendix 2

This supplemental material has been provided by the authors to give readers additional information about their work.

© 2025 Cantor J et al. *JAMA Network Open*.

eFigure 1. Geographic Variation in Availability of an OTP Within a 15-Minute Drive

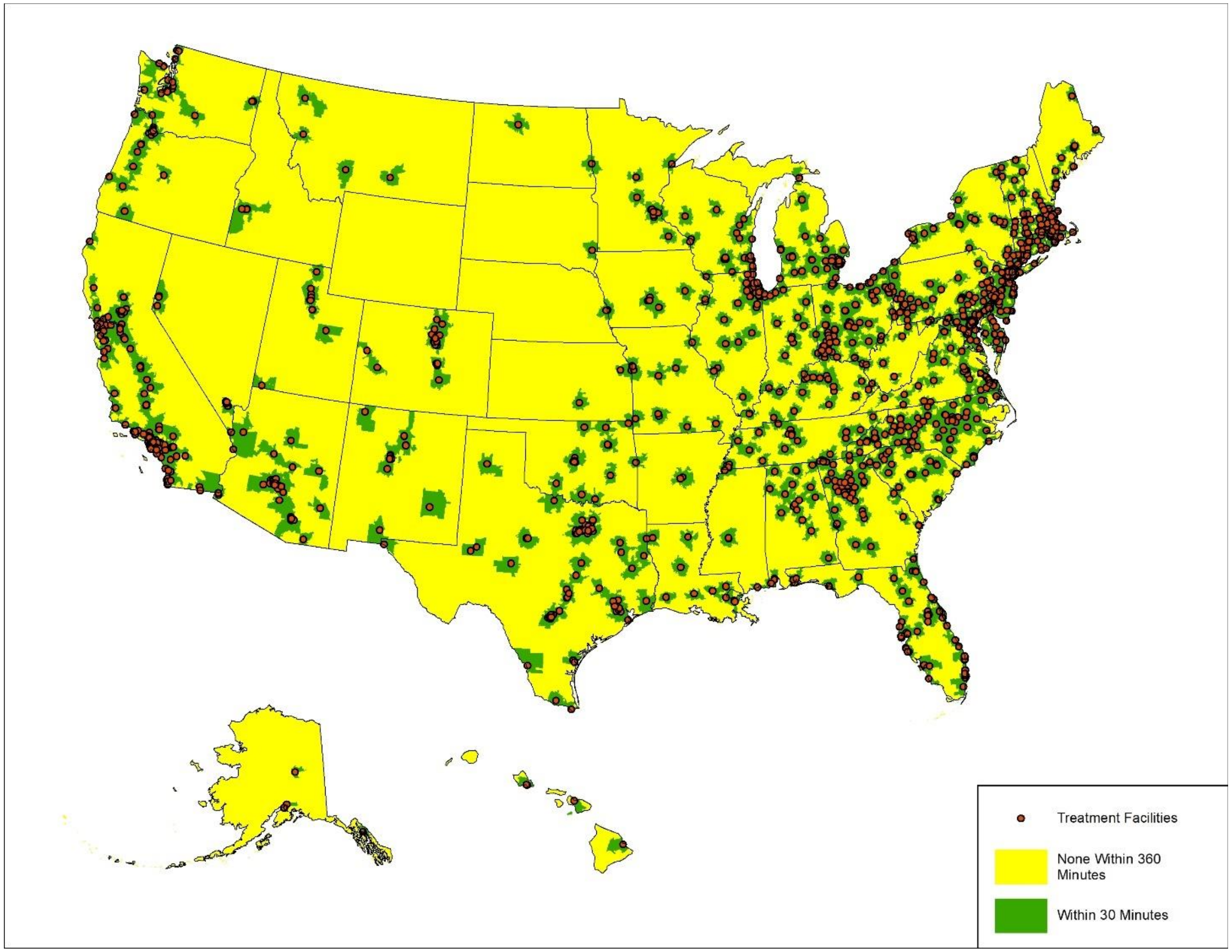

Note: Dots are OTPs that are listed with CMS. Areas in yellow are a ZIP code that lacks an OTP within a 15-minute network drive time from the centroid of the ZIP code. Areas in green are a ZIP that includes an OTP within a 15-minute network drive time.

© 2025 Cantor J et al. *JAMA Network Open*.

eFigure 2. Geographic Variation in Availability of an OTP Within a 30-Minute Drive

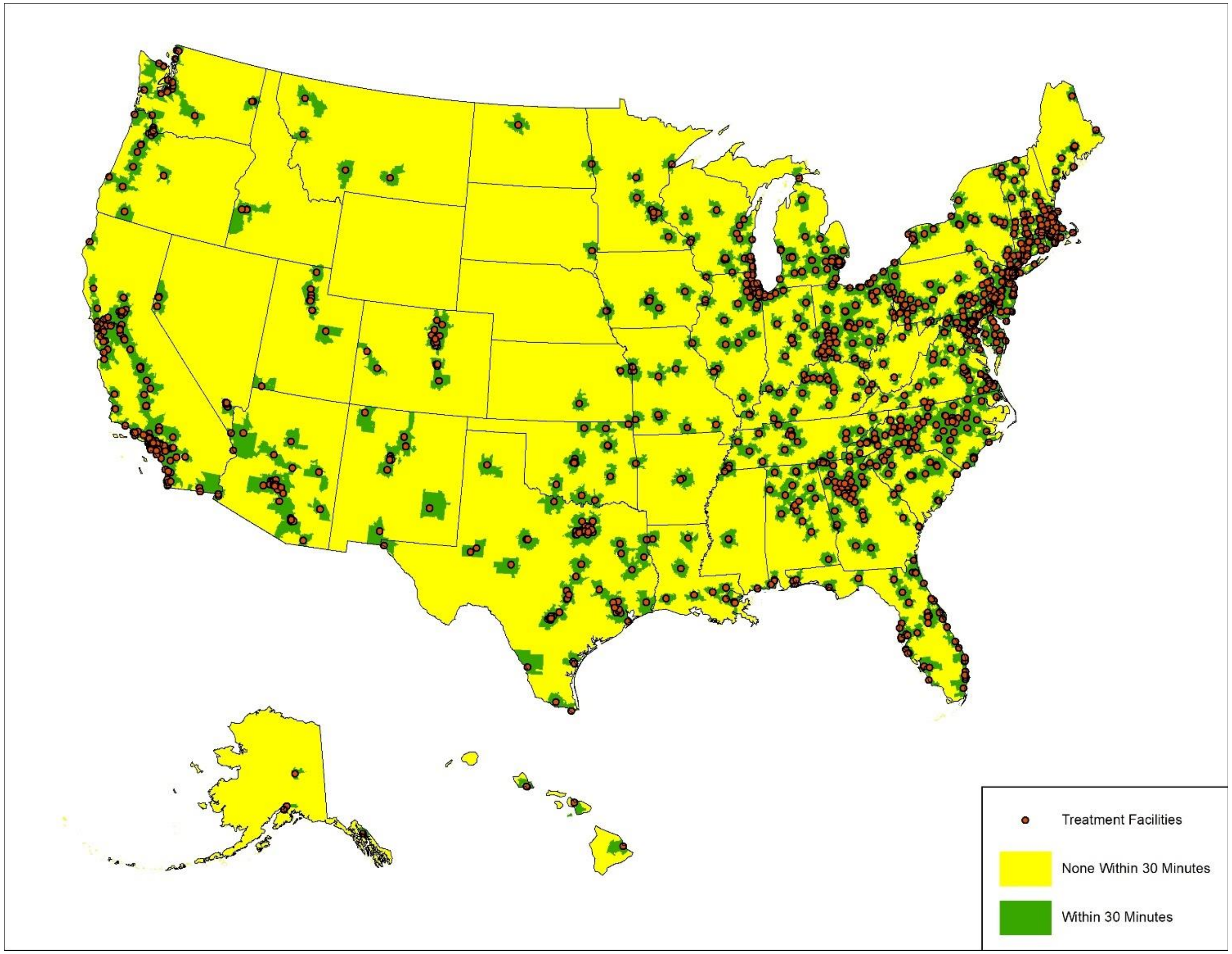

Note: Dots are OTPs that are listed with CMS. Areas in yellow are a ZIP code that lacks an OTP within a 30-minute network drive time from the centroid of the ZIP code. Areas in green are a ZIP that includes an OTP within a 30-minute network drive time.

© 2025 Cantor J et al. *JAMA Network Open*.

eFigure 3. Estimated Probabilities of MOUD Use Based on Drive Time in Each Quarter of 2020

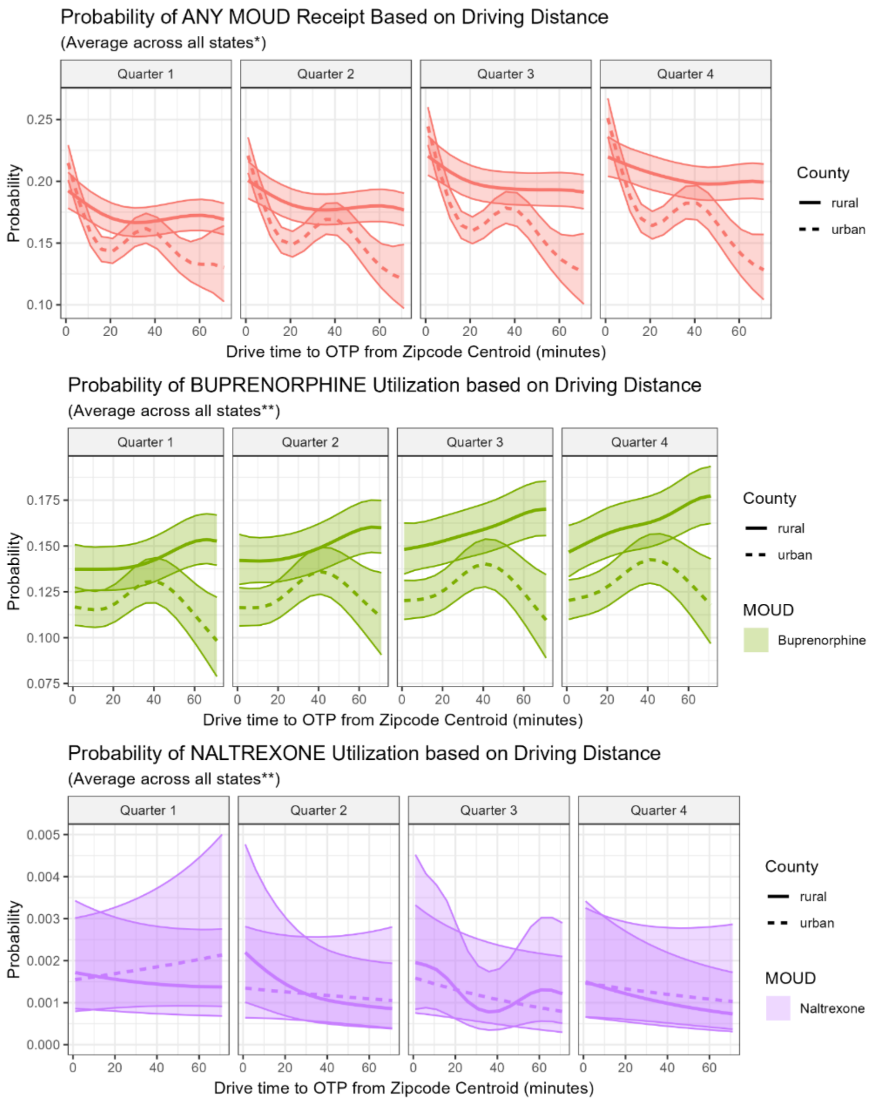

© 2025 Cantor J et al. *JAMA Network Open*.

eTable 1. Empirical National MOUD Receipt Likelihood by Quarter and Drive Time (Minutes) From Nearest OTP Based on a Beneficiary's Zip Code Centroid

	Drive Time	*Any MOUD N(%)*	*Buprenorphine N(%)*	*Naltrexone N(%)*
Quarter 1 (N = 478,820)	< 15 min	46927(9.8)	32322 (6.8)	546 (0.1)
	15-30 min	18078 (3.8)	15088 (3.2)	242 (0.1)
	30-60 min	12981 (2.7)	11519 (2.4)	131 (0.0)
	> 60 min	6721 (1.4)	6062 (1.3)	70 (0.0)
Quarter 2 (N = 459,909)	< 15 min	46736 (10.2)	31468 (6.8)	431 (0.1)
	15-30 min	18109 (3.9)	14843 (3.2)	169 (0.0)
	30-60 min	13042 (2.8)	11466 (2.5)	82 (0.0)
	> 60 min	6700 (1.5)	5995 (1.3)	41 (0.0)
Quarter 3 (N =447,204)	< 15 min	49865 (11.2)	31784 (7.1)	469 (0.1)
	15-30 min	18884 (4.2)	15079 (3.4)	165 (0.0)
	30-60 min	13407 (3.0)	11663 (2.6)	71 (0.0)
	> 60 min	6956 (1.6)	6129 (1.4)	53 (0.0)
Quarter 4 (N = 428,984)	< 15 min	49211 (11.5)	30817 (7.2)	403 (0.1)
	15-30 min	18563 (4.3)	14632 (3.4)	168 (0.0)
	30-60 min	13055 (3.0)	11290 (2.6)	71 (0.0)
	> 60 min	6844 (1.6)	6000 (1.4)	36 (0.0)

© 2025 Cantor J et al. *JAMA Network Open*.

eTable 2. National Likelihood of Any MOUD Receipt by Quarter and Drive Time (Minutes) From the Nearest OTP Based on a Beneficiary's Zip Code Centroid

Drive Time	Quarter	Estimated Likelihood of Rural Utilization (Average of 95% Confidence Interval Bounds Across U.S. States)	Estimated Likelihood of Urban Utilization (Average of 95% Confidence Interval Bounds Across U.S. States)
5	1	18.69 (17.42-20.04)	18.91 (17.7-20.18)
5	2	19.59 (18.27-20.99)	19.64 (18.39-20.95)
5	3	21.51 (20.1-22.98)	21.6 (20.28-22.98)
5	4	21.67 (20.25-23.17)	22.28 (20.92-23.7)
15	1	17.48 (16.3-18.72)	14.66 (13.67-15.7)
15	2	18.52 (17.28-19.82)	15.41 (14.38-16.5)
15	3	20.33 (19.02-21.71)	16.68 (15.59-17.82)
15	4	20.98 (19.63-22.4)	17.24 (16.12-18.42)
30	1	16.66 (15.53-17.85)	15.69 (14.59-16.86)
30	2	17.73 (16.54-18.98)	16.11 (14.98-17.3)
30	3	19.52 (18.26-20.85)	17.16 (15.99-18.4)
30	4	20.2 (18.89-21.58)	17.44 (16.25-18.7)
60	1	17.25 (16.03-18.54)	13.28 (11.49-15.29)
60	2	18.01 (16.74-19.35)	13.19 (11.55-15.03)
60	3	19.31 (17.99-20.7)	13.79 (12.04-15.75)
60	4	19.94 (18.58-21.38)	14.27 (12.53-16.22)

© 2025 Cantor J et al. *JAMA Network Open*.

eTable 3. Empirical State-Specific MOUD in 2020

State	Any MOUD	Buprenorphine	Naltrexone
	N (%)	N (%)	N (%)
Alabama	2823 (24.5)	2340 (20.3)	* (*)
Alaska	312 (21.6)	271 (18.8)	12 (0.83)
Arizona	1493 (9.1)	1068 (6.5)	24 (0.15)
Arkansas	690 (11.5)	618 (10.3)	* (*)
California	10869 (17.2)	5916 (9.4)	55 (0.09)
Colorado	1162 (12.4)	883 (9.4)	15 (0.16)
Connecticut	2673 (35.0)	1289 (16.9)	28 (0.37)
Delaware	669 (12.4)	477 (8.8)	12 (0.22)
District of Columbia	509 (29.4)	403 (23.2)	* (*)
Florida	4223 (8.1)	3640 (7.0)	34 (0.06)
Georgia	1648 (9.8)	1181 (7.0)	* (*)
Hawaii	259 (18.9)	171 (12.5)	- (-)
Idaho	492 (10.6)	467 (10.0)	* (*)
Illinois	2338 (15.0)	1717 (11.0)	40 (0.26)
Indiana	2755 (19.2)	2306 (16.1)	47 (0.33)
Iowa	276 (8.4)	183 (5.6)	* (*)
Kansas	276 (5.5)	226 (4.5)	* (*)
Kentucky	4689 (25.3)	4117 (22.2)	69 (0.37)
Louisiana	1433 (12.2)	1230 (10.5)	25 (0.21)
Maine	2209 (46.8)	1706 (36.1)	* (*)
Maryland	5362 (32.0)	2587 (15.4)	59 (0.35)
Massachusetts	10290 (47.8)	6999 (32.5)	267 (1.24)
Michigan	5475 (21.2)	3949 (15.3)	85 (0.33)
Minnesota	1429 (15.4)	1043 (11.2)	11 (0.12)
Mississippi	1208 (10.3)	1103 (9.4)	* (*)
Missouri	1437 (14.0)	1209 (11.8)	39 (0.38)
Montana	405 (20.6)	332 (16.9)	* (*)
Nebraska	174 (9.3)	156 (8.3)	* (*)
Nevada	549 (7.4)	366 (4.9)	* (*)
New Hampshire	1671 (41.6)	1363 (34.0)	41 (1.02)
New Jersey	3126 (17.2)	2311 (12.7)	59 (0.32)
New Mexico	1056 (19.2)	830 (15.1)	* (*)
New York	7022 (25.3)	4931 (17.7)	135 (0.49)
North Carolina	4544 (19.9)	3752 (16.5)	97 (0.43)
North Dakota	72 (9.6)	66 (8.8)	- (-)
Ohio	5001 (25.8)	4417 (22.8)	151 (0.78)
Oklahoma	1639 (9.5)	1445 (8.4)	* (*)
Oregon	2002 (19.9)	1602 (15.9)	* (*)

© 2025 Cantor J et al. *JAMA Network Open*.

Pennsylvania	6291 (21.2)	5066 (17.1)	190 (0.64)
Rhode Island	1018 (51.6)	709 (35.9)	* (*)
South Carolina	1340 (17.7)	953 (12.6)	*(*)
South Dakota	82 (14.0)	82 (14.0)	- (-)
Tennessee	3447 (21.4)	2932 (18.2)	52 (0.32)
Texas	2592 (7.9)	2139 (6.6)	* (*)
Utah	856 (15.2)	748 (13.2)	11 (0.19)
Virginia	2578 (23.4)	1940 (17.6)	27 (0.25)
Washington	3724 (23.0)	3183 (19.6)	44 (0.27)
West Virginia	2039 (36.7)	1745 (31.4)	69 (1.24)
Wisconsin	1588 (22.6)	1095 (15.6)	48 (0.68)
Wyoming	130 (18.1)	128 (17.8)	- (-)

[A] Utilization at either at OTPs or covered by Part D at pharmacies

[B] Medicare beneficiaries with recently OUD diagnosis that had a Medicare Part D plan

[C] "*"Counts less than 11 are suppressed per CMS privacy protocol.

[D] "-"States where no one with OUD that got treated

© 2025 Cantor J et al. *JAMA Network Open*.

eAppendix 1

***ICD-9* Codes:**

3040, 30400, 30401, 30402, 3047, 30470, 30471, 30472, 30550, 30551, 30552, 96500, 96501, 96502, 96509, E8500, E8501, and E8502

***ICD-10* Codes:**

F1110, F11120, F11121, F11122, F11129, F1114, F11150, F11151, F11159, F11181, F11182, F11188, F1119, F1120, F11220, F11221, F11222, F11229, F1123, F1124, F11250, F11251, F11259, F11281, F11282, F11288, F1129, T400X1A, T400X1D, T400X1S, T400X4A, T400X4D, T400X4S, T401X1A, T401X1D, T401X1S, T401X4A, T401X4D, T401X4S, T402X1A, T402X1D, T402X1S, T402X4A, T402X4D, T402X4S, T403X1A, T403X1D, T403X1S, T403X4A, T403X4D, T403X4S, T404X1A, T404X1D, T404X1S, T404X4A, T404X4D, T404X4S, T40601A, T40601D, T40601S, T40604A, T40604D, T40604S, T40691A, T40691D, T40691S, T40694A, T40694D,T40694S, X42 , and Y12

Source:

Cohrs AC, Husnul Khotimah DE, Dick AW, et al. Spatial and temporal trends in the diagnosis of opioid-related problems in commercially-insured adolescents and young adults. Prev Med. 2022;163:107194. doi:10.1016/j.ypmed.2022.107194

© 2025 Cantor J et al. *JAMA Network Open*.

eAppendix 2.

The general form of the statistical model used in this study are reflected in the equation below. Our outcome of interest in each model was $Y_{ij}^{(k,q)}$ which is an indicator of whether beneficiary i in state j at quarter q received Any MOUD ($k = 1$), Buprenorphine ($k = 2$), Methadone ($k = 3$), or Naltrexone ($k = 4$) such that $Y_{ij}^{(k,q)} = 1$ indicates receipt and $Y_{ij}^{(k,q)} = 0$ indicates no receipt of such medications. Our main goal was to investigate whether of the likelihood of medication receipt, $\pi_{ij}^{(k,q)} = Prob(Y_{ij}^{(k,q)} = 1)$, varied based on a beneficiary's drive time to an OTP (d_i), the beneficiary's residing state, and their local environment as represented by whether the beneficiary lived in an urban area ($Urban_i = 1$) or rural area ($Urban_i = 0$). To account for non-linear patterns in the probability of receipt based on proximity to an OTP, we fit Generalized Additive Models (GAM). In these models, we estimate distinct non-linear association for beneficiaries in urban areas compared to those in rural settings. This approach acknowledges that geographical barriers, such as drive time, may be experienced differently at a local level.

$$logit\left(\pi_{ij}^{(k,q)}\right) = \beta_0 + \tau_j + \beta_2 \times Urban_i + f_1(d_i \mid Urban_i = 0) + f_2(d_i \mid Urban_i = 1)$$

In the model formulation, τ_j is a state fixed effect to account for differential MOUD receipt rates between states while exponentiating β_2 helps describe the difference in likelihood of medication receipt for urban areas compared to rural areas. Further, $f_1$and f_2 help capture the smooth non-parametric association between proximity to an OTP and the outcome modeled, conditional on whether when a beneficiary is in a rural or urban setting, respectively.
