# Supplementary material for: Medicare Beneficiary Receipt of Methadone by Drive Time to Opioid Treatment Programs
Source: JAMA Netw Open. 2025 Apr 3;8(4):e253099. doi: 10.1001/jamanetworkopen.2025.3099 (PMC11969284; doi:10.1001/jamanetworkopen.2025.3099)
Supplement: Supplement 2. — Data Sharing Statement [file jamanetwopen-e253099-s002.pdf]

Data Sharing Statement

Cantor. Medicare Beneficiary Receipt of Methadone by Drive Time to Opioid Treatment Programs. *JAMA Netw Open*. Published April 03, 2025.
doi:10.1001/jamanetworkopen.2025.3099

Data
Data available: No

Additional Information
Explanation for why data not available: The data has been acquired through a DUA.
